# Editorial: Neural and Epigenetic Factors in Parenting, Individual Differences and Dyadic Processes

**DOI:** 10.3390/brainsci12040478

**Published:** 2022-04-05

**Authors:** Livio Provenzi, Serena Grumi, Maria José Rodrigo

**Affiliations:** 1 Department of Brain and Behavioral Sciences, University of Pavia, 27100 Pavia, Italy; 2 Child Neurology and Psychiatry Unit, IRCCS Mondino Foundation, 27100 Pavia, Italy; serena.grumi@mondino.it; 3 Department of Developmental Psychology and Education, Faculty of Psychology, University of La Laguna, 38200 La Laguna, Spain; mjrodri@ull.es; 4 University Institute of Neuroscience, University of La Laguna, 38200 La Laguna, Spain

Human parenting is a fundamental educational context including complex caregiving tasks finalized to nurture and protect young children [1]. Parenting is seen as comprising a set of capacities, mental health conditions, and cognitive, emotional, motivational, and behavioral dispositions to satisfy the child’s needs that may vary from parent to parent. Parenting is also best understood as a transactional dyadic process between both caregivers as well as parent–child interactions aimed at their co-adaptations [2]. Behavioral evidence shows that the quality of this early sensitive and responsive interaction in daily exchanges is crucially associated with healthy child development [3] and predicts infants’ attachment quality [4]. The biological priming of human parenting makes it a fertile ground for exploring neural and epigenetic factors that shape the complexities of the caregiving dimensions in both at-risk and normative samples. The quality of early caregiving represents a key environment influencing the offspring’s psychophysiological development both in humans [5,6] and nonhuman animals [7,8,9].

Research in this field primarily investigated evidence on the effects that early caregiving adverse experiences have on infants and children [10]. Child maltreatment includes all types of physical and/or emotional ill-treatment, which results in actual or potential harm to the child’s healthy development, survival, or dignity. Therefore, it is expressed in several ways, ranging from the neglect pole and the opposite extreme of overprotection. Previous studies have shown that exposure to maltreatment is linked to brain (e.g., smaller prefrontal cortex volume in both grey and white matter [11]), genetic, and epigenetic (e.g., telomere shortening [12]; *NR3C1* methylation [13]) alterations in children. Focusing on the maternal side, previous studies showed that caregiving capacities to respond to the child’s signals are altered in adverse contexts indexed by brain failures in the early differentiation of cry stimuli and in the sustained processing of infant expressions [14], limbic-visual attenuation in response to infant crying faces as compared to adult faces [15], reduced inferior fronto-temporo-occipital structural connectivity [16], and volume reductions in empathy-related areas [17].

As the field of behavioral epigenetics and neuroscience moves toward incorporating the study of protective exposures—and not only adversities and stressful encounters—the investigation of normative caregiving contexts is also prominent. Indeed, animal model studies confirmed that variations in the normative range of caregiving behaviors (e.g., pup licking and grooming and arched-back nursing in rats) also altered the offspring epigenome [18]. However, the complexity of human caregiving poses more challenges in exploring its risk or protective impact on children’s neuropsychobiological development.

From this perspective, the present Special Issue addressed such complexity by focusing on individual differences and dyadic processes considering both dysfunctional and typical caregiving pathways. It aimed at collecting evidence derived from cutting-edge neuroimaging techniques and behavioral epigenetics. The collection includes original research articles and systematic reviews from researchers and clinicians working in different European (i.e., Spain, Belgium, Italy, Netherlands, Finland, England), North American (i.e., Illinois, Michigan, Colorado), Asiatic (i.e., Singapore), and African (i.e., South Africa) countries. The seven papers included in this issue vary in terms of subjects, mechanisms, and investigated outcomes, but can be categorized in two groups of studies addressing the neuropsychobiology of caregiving in risk conditions or in normative contexts.

Original articles addressing dysfunctional parenting have focused on neglectful caregiving and its neural and epigenetics and on genetic vestiges of overprotecting parenting. Comparing maternal neglectful and non-neglectful caregiving, Leon and colleagues [19] investigated cortical differences (i.e., thickness and surface area) and their relations with the dyadic emotional availability, whereas Herrero-Roldán and colleagues [20] tested the epigenetic age acceleration and potential protective factors, such as empathic concern, that may reduce this health vulnerability and the consequent poor social functioning in neglectful caregiving. The study performed by Bonassi et al. [21] further shed light on the long-term consequences of early life stressors (i.e., overprotecting parenting) on adult social life through a well-established gene environment perspective.

Besides extreme adverse circumstances, such as child maltreatment, human research on the influence of the environment is only beginning. Learning the neural and epigenetic basis of caregiving and how variations in parenting in the normal range affect the brain development and psychophysiological functioning of children not exposed to extreme adversity is of vital importance [22]. Original articles and reviews focused on normative parenting in the present collection included the study of neuropsychobiology of both mothers and fathers. The review performed by Clark et al. [23] adopted the emotional availability framework as a promising window to investigate how stress physiology, neuroendocrine system, genetics, and epigenetics may be associated with adults’ and children’s brain development and intergenerational transmission of specific genetic and neurobiological markers. Since epigenetic effects have been reported in literature in both adults’ and children’s problematic behavior, this raises the question of whether parents and children could have similar levels of methylation of stress-regulation-related genes. Therefore, Van Aswegen et al. [24] tested the convergence of mother and child epigenetic patterns in families, and their findings at least partially supported that child and parent methylation levels covary. 

Another topic explored in neuropsychobiological studies regards the transition to parenthood in mothers and fathers. As for mothers, brain changes (e.g., grey matter volume reduction) linked to the maternal attachment towards their baby have been largely investigated in the first two years after delivery, although no studies were available about long-term modifications. Martínez-García et al. [25] addressed this gap, exploring the grey matter volume reduction six years after childbirth and their preliminary findings open the possibility that pregnancy-induced brain changes are permanent. Recently, understanding the neurological underpinnings of fathering has become a key research issue in developmental psychobiology studies, given that fathers are increasingly involved in childcare. The study by Provenzi et al. [26] provides a review of functional magnetic resonance imaging (fMRI) studies conducted so far on the neurological correlates of fatherhood and paternal caregiving in humans with a specific focus on brain responses to infant-related stimuli. Preliminary evidence suggested that paternal caregiving behaviors may only partially rely on the same neural circuits and networks linked with maternal caregiving.

Taken together, these studies highlight the relevance of deepening our knowledge about human caregiving and the progressive methodological advances that allow us today to investigate the underling neurobiological processes of parenting. The reviews and the original papers collected in this Special Issue further contribute by providing relevant inputs for both clinical and translational research. Future advances in these areas will greatly contribute to our comprehension of the neuropsychobiology of parenting in typical and at-risk conditions, and hopefully, they will take part in improving our capacity to care for infants and their parents by developing effective and efficient preventive and therapeutic strategies.
